# Genome sequence of the *Klebsiella quasipneumoniae* bacteriophage EKq1 with activity against *Klebsiella pneumoniae*

**DOI:** 10.1128/MRA.00954-23

**Published:** 2023-11-30

**Authors:** Jordan T. Bird, Kevin A. Burke, Caitlin D. Urick, Jamie L. Braverman, Nino Mzhavia, Damon W. Ellison, Mikeljon P. Nikolich, Andrey A. Filippov

**Affiliations:** 1 Department of Biochemistry and Molecular Biology, University of Arkansas, Little Rock, Arkansas, USA; 2 Wound Infections Department, Bacterial Diseases Branch, Walter Reed Army Institute of Research, Silver Spring, Maryland, USA; 3 Bacterial Diseases Branch, Walter Reed Army Institute of Research, Silver Spring, Maryland, USA; DOE Joint Genome Institute, Berkeley, California, USA

**Keywords:** *Klebsiella quasipneumoniae*, *Klebsiella pneumoniae*, phage EKq1, complete genome sequence, class Caudoviricetes, unclassified Caudoviricetes, lytic phage, therapeutic candidate

## Abstract

We describe the genome of a lytic phage EKq1 isolated on *Klebsiella quasipneumoniae*, with activity against *Klebsiella pneumoniae*. EKq1 is an unclassified representative of the class Caudoviricetes, similar to *Klebsiella* phages VLCpiS8c, phiKp_7-2, and vB_KleS-HSE3. The 48,244-bp genome has a GC content of 56.43% and 63 predicted protein-coding genes.

## ANNOUNCEMENT

Phages are used as alternative or adjunct antibacterials against multidrug-resistant (MDR) *Klebsiella pneumoniae* infections (1 – 3). *K. pneumoniae* phages usually have limited host ranges (4), but phages with broader activity can be isolated on near-neighbor species (5, 6). Here, we describe the genome of phage EKq1 isolated on *Klebsiella quasipneumoniae* and capable of lysing some MDR *K. pneumoniae* clinical isolates.

EKq1 was isolated from sewage collected on 16 March 2022, in Montgomery County, Maryland, using a human blood isolate, *K. quasipneumoniae* MRSN 829456, for phage enrichment. The enrichment was performed as described (7), in broth with shaking at 37°C, and phage plaques were detected on double-layer agar plates. The phage was purified by three single plaque isolations, and its DNA was extracted from lysate with the QIAamp DNA Mini Kit (Qiagen, Germantown, MD), per the manufacturer’s protocol. A library was constructed using the KAPA HyperPlus Kit (Roche Diagnostics, Indianapolis, IN) and sequenced on an Illumina MiSeq (Illumina, San Diego, CA) with a 600-cycle MiSeq Reagent Kit v3 that produced 300-bp paired-end reads. Paired-end sequences (1,864,294 reads total) were assessed for quality using FastQC 0.11.9 (8) and trimmed with Trimmomatic (9) v0.39. Phage EKq1 genome was assembled *de novo* using Unicycler 0.4.8 (10), its termini and DNA packaging mechanism were determined using PhageTerm (11), and lifestyle was predicted using BACPHLIP (12). Protein-coding sequences (CDSs) were annotated using the Pharokka pipeline (13 – 23). Amino acid sequence similarity searches were performed in Diamond (24, 25) against the nr database downloaded in January 2023. All tools were run with default parameters.

**Fig 1:**
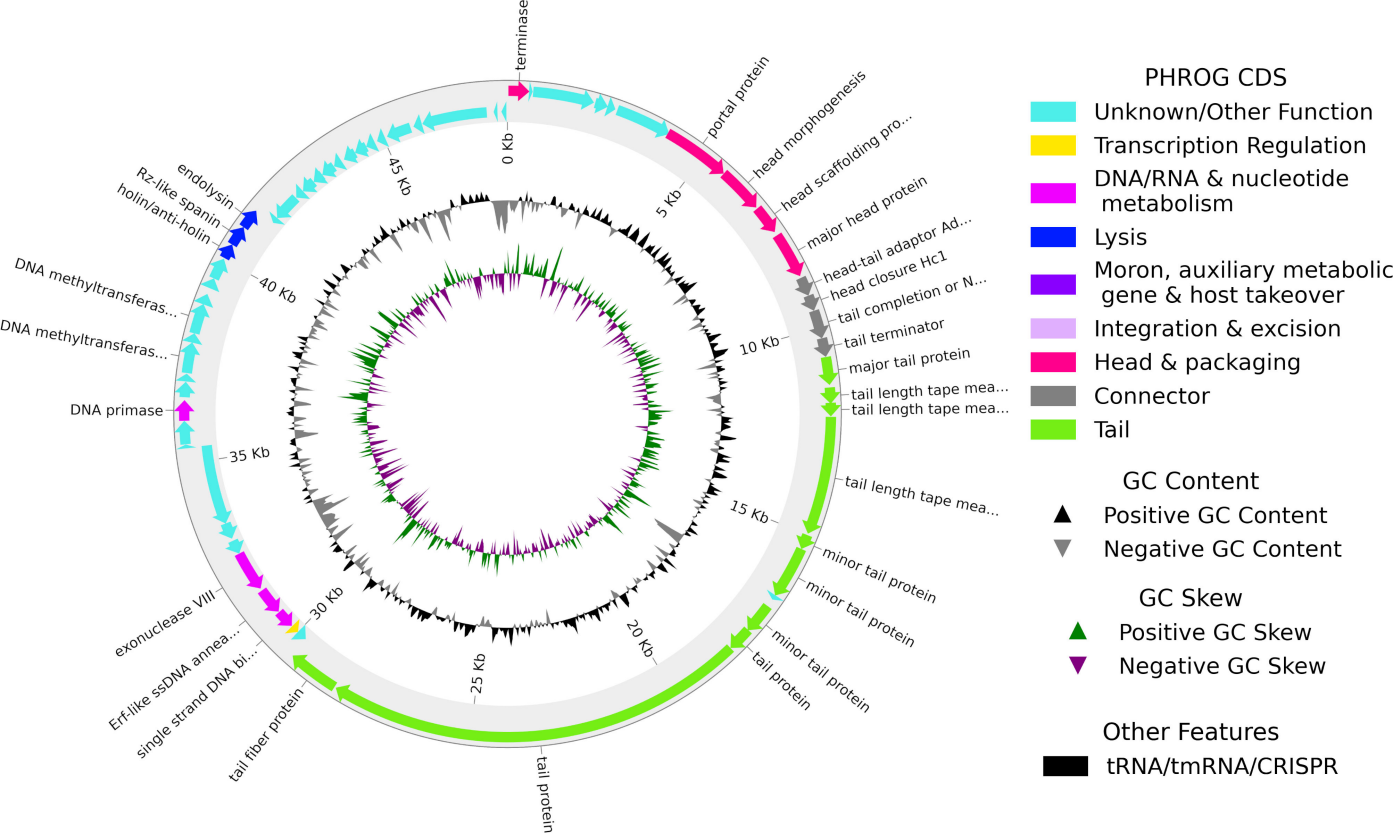
Genome organization of EKq1.

The average read coverage was 977×; EKq1 genome was 48,244 bp long, with G + C content of 56.43%, contained 63 predicted CDSs (Fig. 1) and direct terminal repeats of 8,798 bp. Mash alignment (23) to the INPHARED database (22) placed EKq1 among *Klebsiella* siphophages currently classified as class Caudoviricetes, with highest DNA identity, >98%, to VLCpiS8c (GenBank ON602734), phiKp_7-2 (LC768468), and vB_KleS-HSE3 (26; MT075871). Phage vB_KleS-HSE3 was lytic against one of four tested MDR *K. pneumoniae* strains (26). Nucleotide identity higher than 95% indicates that phages EKq1, VLCpiS8c, phiKp_7-2, and vB_KleS-HSE3 belong to the same species (27). These phages contain a putative Cas4-like exonuclease. A similar exonuclease in *Campylobacter* phages stimulated acquisition of host-derived spacers by the bacterial CRISPR-Cas system that might be a decoy to prevent phage DNA acquisition and, therefore, an anti-CRISPR measure (28).

BACPHLIP scored EKq1 genome at 89%, while the threshold for high-confidence lytic lifestyle is 95% (12). Additionally, nucleotide BLAST search (29) against the nr database found a region of EKq1 DNA with 78%–89% identity to bacterial chromosomes, within prophage genes (e.g., MKK01_09025 and MKK01_09030 in *Klebsiella variicola*, GenBank CP092632). However, these genes encode a carbohydrate-binding domain protein and a tail fiber protein and have no relation to lysogenicity. EKq1 putative proteins showed no homology to products related to lysogenic lifestyle, gene transfer, and bacterial proteins including antibiotic resistance determinants (18) and virulence factors (19). Thus, EKq1 appears to be a lytic phage and a candidate for therapeutic use.

## Data Availability

The EKq1 genome BioProject, BioSample, GenBank, and the NCBI Sequence Read Archive accession numbers are PRJNA1016341, SAMN37380043, OR555718, and SRR26063729, respectively.
